# Caregivers in amyotrophic lateral sclerosis (ALS): preliminary results between paid care and family care

**DOI:** 10.1080/21642850.2026.2709184

**Published:** 2026-09-19

**Authors:** Elisa Zambetti, Irene Baronchelli, Simone Belli, Clara Mucci, Andrea Greco

**Affiliations:** a Dipartimento di Scienze Umane e Sociali, Università degli Studi di Bergamo; b Villa Dei Cedri – Residenza Sanitaria Assistenziale (Merate, LC); c Departamento de Antropología Social y Psicología Social, Facultad de Ciencias Políticas y Sociología, Universidad Complutense de Madrid, Spain; d Universidad ECOTEC, Samborondón, Ecuador

**Keywords:** Formal and informal caregivers, paid or family care, psychological well-being, amyotrophic lateral sclerosis, mixed-method research

## Abstract

**Background:**

Amyotrophic lateral sclerosis (ALS) is a rare neurodegenerative disease affecting about 6,000 people in Italy. Caring for ALS impacts the quality of life and mental health of formal and informal caregivers, but few studies have explored this.

**Methods:**

This research analyzes experiences of 9 formal caregivers (FC; 8 women) with an average age of 49.29 years (*SD =* 3.861) and 6.58 years of average length of service (*SD =* 5.975), and 11 informal caregivers (iFC; 9 women) with an average age of 48.30 years (*SD =* 13.500) and who have been caring for relatives for 3.29 years (*SD =* 2.500). All participants were recruited from an Italian healthcare residence. We assessed psychological well-being, burnout (for FC), and burden (for iFC), and included testimonies retrieved from online sources. Data were analyzed using mixed-methods analysis, including nonparametric tests, Emotional Text Mining, and discourse analysis.

**Results:**

The results showed both groups had high psychological well-being (*Mfc* = 85.86; *SDfc* = 9.026; *Mifc* = 83.10; *SDifc* = 11.435). FC had low emotional exhaustion (*M =* 16.67; *SD =* 10.087) and depersonalization (*M =* 2.56; *SD =* 2.186), and a moderate level of personal gratification (*M =* 29.11; *SD =* 11.667; *M =* 27.43; *SD =* 11.238). iFC experienced a higher burden when the patient is in a healthcare residence than when he/she was at home. FC discussed patient and family challenges, emphasizing the necessity of research and new therapies; iFC focused on social and emotional needs, seeing themselves as adaptors out of necessity.

**Conclusions:**

The study highlights the psychological impact of caregiving and preliminary qualitative differences between paid and family caregivers. Further analysis could explore these distinctions, aiding the development of targeted interventions to promote caregivers’ well-being.

## 1. Introduction

Amyotrophic lateral sclerosis (ALS), also known as motor neuron disease (MND), is characterized as a rare, heterogeneous, multisystem progressive neurodegenerative disorder with an unknown and clear etiology (Mitchell & Borasio, 2007). Globally, incidence and prevalence are estimated at two to four cases per 100,000 individuals and approximately six individuals per 100,000, respectively – an increasing figure (Gordon, 2011, 2013; Mitchell & Borasio, 2007). In Italy, the most recent data, updated in February 2020 and published by the Italian Association for Amyotrophic Lateral Sclerosis (AISLA), based on prevalence statistics from the European Consortium for Amyotrophic Lateral Sclerosis (EURALS Consortium), indicate that over 6,000 individuals are affected by ALS, with an annual increase of about 2,000 cases.

ALS involves motor neurons and is now understood to involve other affected areas, e.g. the limbic system. Its causes are unclear, but researchers identify three risk factors: genetic mutations and a familial history (5% of cases), being male (1.5:1 ratio), and advancing age (Abdulla et al., 2014; Ingre and Roos, 2015; Kiernan et al., 2011; Kuraszkiewicz et al., 2018; Lang et al., 2020; Lillo et al., 2012; Logroscino et al., 2010; Mezzapesa et al., 2007). Investigations of environmental and social risk factors show conflicting evidence. There is some evidence that shortened telomere length, seen in high-stress environments, may increase susceptibility to ALS in adulthood. These findings indicate that adverse experiences during early life may contribute to ALS susceptibility. Research indicates a link between adverse childhood experiences (ACEs) and later diseases. However, further research is needed to understand the connection between early nervous system disorders and ALS (Felitti et al., 1998).

ALS initially presents with non-specific symptoms such as muscle weakness, fatigue, fasciculations, cramps, and speech alterations, often delaying diagnosis (Hardiman et al., 2011). As the disease progresses, motor impairment leads to widespread disability, including dysarthria, dysphagia, sialorrhea, pain, constipation, and severe respiratory failure in advanced stages. Patients experience increasing fatigue and functional decline across the early, mid, and advanced stages, requiring assistive devices and eventually palliative care. Beyond physical symptoms, ALS also has profound psychological consequences, including anxiety, depression, existential distress, and reduced quality of life throughout the disease course stages (Gordon, 2011, 2013; Mitchell & Borasio, 2007; Vucic et al., 2014; Zucchi et al., 2019). Due to its unique characteristics, absence of a definitive cure, and rapid progression, ALS necessitates 24 h care. In the most cases, assistance is givenin the home context; but, sometimes, it happens that in the last stages ALS patients within healthcare facilities, significantly impacting the quality of life (QoL) of patients and their formal and informal caregivers (namely, family members and all those who care for patients but do not receive financial compensation because this is not their job) (Conroy et al., 2023). Chronic disabilities profoundly influence family life, disrupting internal dynamics and affecting the psychological and social well-being of family members (Glozman, 2004), and also the practical organization and structure of the family. These effects are not solely attributable to the clinical features requiring caregiver support but also include relational dynamics. Interactions with the external environment often diminish, elevating the importance of familial bonds, which in turn influence the perception of QoL (Glozman, 2004). The World Health Organization (WHO) defines (World Health Organization, 1995) QoL as ‘​​​​​​*an individual’s perception of their position in life in the context of the culture and value systems in which they live and in relation to their goals, expectations, standards, and concerns.*’ (p. 1405). Despite ongoing debates, this definition is widely accepted, encompassing six critical domains: physical well-being, psychological well-being, independence, social relationships, environmental factors, and personal beliefs/spirituality.

Chronic diseases, particularly ALS, therefore impact caregivers’ psychological and physical health, especially when patients require placement in a residential or semi-residential healthcare residence (Conroy et al., 2023). Here, medical and social healthcare workers are tasked with addressing patient needs, transitioning caregivers into formal roles recognized within institutional frameworks, and being compensated accordingly (Poppe et al., 2022). Consequently, the well-being and QoL of caregivers, especially those providing formal and paid care, are often compromised due to the demands of sustained assistance (Karabuga Yakar et al., 2023).

Numerous studies have demonstrated that burnout, a syndrome characterized by chronic workplace stress, as defined by the WHO in the ICD-11, affects caregivers and healthcare professionals, with a higher prevalence among women (Baiocco et al., 2004; Caruso et al., 2011; Sirigatti & Stefanile, 1993; Tartaglia & Vannucci, 2013). Conflicting evidence exists regarding the relationship between years of professional experience and burnout risk; some research indicates that younger caregivers are more vulnerable due to less developed coping strategies (Molero Jurado et al., 2018), whereas other studies suggest that more experienced caregivers are equally or more at risk, owing to cumulative stress (Pradas-Hernández et al., 2018). The presence of children or a family unit appears to serve as a protective factor (Calamandrei & Orlandi, 2002).

In the context of ALS, assessments of caregivers’ (both paid and familiar) psychological well-being remain insufficient; thus, a comprehensive investigation is crucial to developing effective support strategies. Additionally, understanding the themes emerging from caregiver narratives is essential, yet limited studies have explored formal caregivers’ perspectives on their experiences and emotions in caring for ALS patients (De Wit et al., 2018). Concerning paid care, the few studies that have been conducted show that formal caregivers’ testimonies mainly highlight priorities for patients and families (Poppe et al., 2022). Concerning family care, research shows that key issues concern uncertainties regarding their ‘new life’ and the progression of the disease until death, and feelings of injustice concerning their imposed circumstances with the disease and experience both physical and psychological loneliness (Grabler et al., 2018; Ozanne et al., 2013). In addition, family members’ themes are related to the significance of the disease, personal life alterations, family dynamics, societal relationships, as well as the need for practical and psychological support (Bolmsjö & Hermerén, 2001; Cipolletta & Amicucci, 2015; De Wit et al., 2018; Yang et al., 2024). Overall, there is a significant gap in knowledge regarding the personal narratives and emotional experiences of formal and informal caregivers involved in ALS paid or family.

## 2. Aims

We conducted this research using an interdisciplinary and mixed-methods approach, building on previous studies and literature on these issues. Our focus was on understanding the quality of life of caregivers, to increase understanding of the psychological aspects that have been less thoroughly analyzed in the literature, so that future interventions proposed to caregivers can promote their well-being and reinforce their psychological resources. This study examines the impact of amyotrophic lateral sclerosis (ALS) from an interdisciplinary perspective to understand the psychosocial factors influencing healthcare workers and family members. We hypothesize that caregivers experience low levels of well-being alongside high levels of stress, burnout, and burden, and we wanted to compare family members before and after patient acceptance of a healthcare residence. We also aim to explore how ALS is perceived by healthcare professionals and family members, identifying support needs. We analyze themes to highlight the importance of understanding ALS beyond its medical aspects. In general, our study aims to explore and conduct a preliminary analysis of the variables listed.

## 3. Methods

This study employed a mixed-methods design, combining quantitative data analysis to identify generalizable patterns with qualitative analysis to capture subjective experiences and contextual factors. The integration of both approaches strengthened the analysis and reduced the limitations of using a single method.

Based on a narrative literature review, the research was structured into two phases. The first involved formal and informal caregivers in a nursing home, following initial observations and participant recruitment. The second involved online testimonials, adopted as an alternative to interviews that could not be sustained with the initial participants.

### 3.1. Villa dei Cedri’s formal and informal caregivers

The first part of this study was carried out in collaboration with Villa dei Cedri nursing home in Merate (LC), an institution accredited by the Lombardy Region since 2003. The facility offers 120 beds for non-self-sufficient elderly individuals, including those affected by Alzheimer’s disease, dementia, ALS, and/or other neurodegenerative conditions. According to its official online description, Villa dei Cedri is located within a park of over 130,000 square meters and is designed with the comfort and organization of a hotel complex, where healthcare professionals and medical staff provide continuous assistance. Among the various services, the facility includes a Chapel, offering residents a space for devotion, reflection, and prayer. The ALS unit specifically includes 14 dedicated beds, each equipped with centralized oxygen therapy and bedside suction devices. The healthcare professionals working in the facility are carefully selected and specifically trained to care for individuals with complex neurodegenerative diseases. Care delivery is based on a collaborative and multidimensional approach that emphasizes personalization of care relationships, actively involving family members in the development of individualized care plans. Clarifying this research context is essential, as it aids the interpretation of the findings derived from data analysis. The study received ethical approval from the Lombardy Territorial Ethics Committee 2.

Thanks to Villa dei Cedri’s collaboration, we involved a total of 20 participants: 9 were healthcare workers (or formal caregivers), and 11 were family members (or informal caregivers). They were all recruited voluntarily, all the formal caregivers operate in the ALS unit, and all the family members had relatives living in Villa dei Cedri. Regarding formal caregivers, there was only 1 male, the age range was from 44 to 53 years (*M =* 49.29; *SD =* 3.861), and the average length of service (that is, the average number of years that the healthcare worker has been employed at Villa dei Cedri) was 6.58 years (*SD =* 5.975). Different professionals were involved, including Nurse, Physician, Physiotherapist, Psychologist, and Occupational Health Worker (OSS)t^1^. Regarding family members, there were only 2 males and one questionnaire was excluded because of its unreliability, the age range was from 29 to 74 years (*M =* 48.30; *SD =* 13.500), and the average length of been caring for relatives was 3.29 years (*SD =* 2.500)^2^. Table 1 shows the sociodemographic characteristics of operators and family members. To be included in the study, formal caregivers had to work in a unit and team dealing with people diagnosed with ALS, and informal caregivers had to have a relative living in the ALS Unit of Villa del Cedri. All participants had to understand the Italian language. There were no exclusion criteria.

**Table 1.** Sociodemographic characteristics of participants.

	Formal caregivers (Operators)		Informal caregivers (Family members)	
	N	%	N	%
**Gender**				
Male	1	11.1	1	10.0
Female	8	88.9	9	90.0
**Relationship status**				
Married	4	44.4	6	60.0
Divorced/Separated	4	44.4	3	30.0
Single	1	11.1	1	10.0
**Educational level**				
≤8 Years	0	0.0	1	10.0
>8 Years	9	100.0	9	90.0
**Parental status**				
Son/Daughter	/	/	6	60.0
Spouse	/	/	2	20.0
Brother/Sister	/	/	1	10.0
Close friend	/	/	1	10.0

All participants provided informed consent to participation and the use of anonymized data before completing the questionnaire battery. Those who accepted the conditions were granted access. Formal caregivers completed the questionnaire battery online via Google Forms, while informal caregivers completed it on paper.

The first questionnaire, administered to both formal and informal caregivers, was the Psychological Well-Being Scale (PWB-18) (Mirandola et al., 2019; Ruini et al., 2003). It is an 18-item self-report scale using a 6-point Likert scale (1 = *it is not my case* to 6 = *it is just my case*) assessing six dimensions of psychological well-being: autonomy (items 9 reverse, 11, 18), environmental control (items 1, 3 reverse, 6), personal growth (items 7, 15, 17 reverse), positive interpersonal relations (items 4 reverse, 12, 13 reverse), purpose in life (items 5 reverse, 14, 16 reverse), and self-acceptance (items 2, 8, 10 reverse). Each subscale has a maximum score of 18, and a total well-being score (maximum 108) is also calculated. Higher scores reflect greater psychological well-being.

The second questionnaire was the Maslach Burnout Inventory (MBI) (Maslach et al., 1997; Sirigatti & Stefanile, 1993) for formal caregivers and the Caregiver Burden Inventory (CBI) (Greco et al., 2017; Novak & Guest, 1989) for informal caregivers. MBI is a 22-item self-report questionnaire using a 7-point Likert scale (0 = *never* to 6 = *every day*) assessing three independent burnout dimensions: emotional exhaustion, i.e. a negative experience of emotional coldness and disinterest in one’s work and others (items 1, 2, 3, 6, 8, 13, 14, 16, 20; maximum score 54), depersonalization, i.e. a negative experience of enacting cold and impersonal responses and behaviors toward users (items 5, 10, 11, 15, 22; maximum score 30), and personal gratification, i.e. the positive perception related to one’s competence and desire for success in working with others (items 4, 7, 9, 12, 17, 18, 19, 21; maximum score 48). High scores on emotional exhaustion (≥30) and depersonalization (≥12), and low scores on personal accomplishment (≤33) indicate a high risk of burnout in the specific dimension. Moderate risk is defined as scores between 18 and 29 for emotional exhaustion, 6–11 for depersonalization, and 34–39 for personal accomplishment. Low risk is defined as ≤17 for emotional exhaustion, ≤5 for depersonalization, and ≥40 for personal accomplishment. CBI is a 24-item self-report questionnaire on a 5-point Likert scale (0 = *low* to 4 = *high*) designed to assess the level of burden experienced by caregivers. A total burden score (maximum 100) is calculated by summing all item scores, with higher scores indicating a greater caregiving burden. In addition, the CBI measures five specific dimensions (each with a maximum score of 20): time-dependent burden, i.e. the burden associated with time restrictions (items T1–T5), developmental burden, i.e. feelings of being cut off from peers’ opportunities and life stages (items S6–S10), physical burden, i.e. chronic fatigue and health-related issues (items F11–F14), social burden, i.e. perceived role conflicts (items D15–D18), and emotional burden, i.e. emotional reactions toward the care recipient (items E20–E24). For each dimension, a mean score can also be calculated, and if it is greater than 2.5, it indicates an excessive level of burden (Caserta et al., 1996; Novak & Guest, 1989). In order to compare the extent of the burden on family members before and after admission to the facility, we administered, as part of the same set of questionnaires, a past-oriented version of the CBI and a present-oriented version. Specifically, the two instructions for completing the questionnaire were, respectively, as follows:

Section 1 of the questionnaire (CBI related to the present)

*The questions below refer to you as the caregiver for your sick relative.*

*Thinking about the CURRENT SITUATION, please indicate – by choosing a number from 0 to 4 – how often these situations have occurred, by checking the box that best matches your condition or personal impression.*

Section 3 of the questionnaire (CBI referring to the past)

*We will now repeat the first questions, which refer to you caring for your sick relative, but this time in relation to a past situation.*

*Thinking now about the PAST SITUATION, when you had to care for your family member with ALS who was still living at home, please indicate, by choosing a number from 0 to 4, how often these situations occurred, by checking the box that best matches your situation or personal impression.*

At the end of all questionnaires, a series of sociodemographic questions was included to collect information on formal and informal caregivers, specifically regarding age, gender, education level, marital status, length of service (for formal caregivers) or home caring (for informal caregivers), role within the team and service department (only for formal caregivers), and parental status with ALS patient (only for informal caregivers).

Given the small sample size, nonparametric statistical analyses were performed using the Kruskal-Wallis test for independent samples and correlation analyses, using SPSS software (version 29.0.1.0).

### 3.2. Online testimonies

For the second part of this study, 118 narratives written in Italian and collected from various online sources (e.g. association websites, blogs, magazines, and newspapers) were analyzed. Among these, 10 narratives were produced by formal caregivers providing paid care for ALS patients, and 41 narratives were produced by informal caregivers providing family care for ALS relatives. The remaining narratives were produced by patients with ALS. A mixed-method approach was applied to the entire set of narratives. First, the Emotional Text Mining (ETM) technique (Cordella et al., 2014; Greco, 2016) was employed, using T-Lab software, to obtain an initial quantitative overview of implicit communication axes and emerging thematic patterns^3^. Subsequently, discourse analysis (Ruiz Ruiz, 2009) and positioning analysis (Davies & Harré, 1990) were conducted to qualitatively deepen the understanding of themes and dimensions related to ALS, integrating them within the broader social context in which they were produced. The combined findings of these analyses, including results from the three population groups (patients, formal caregivers, and informal caregivers) that comprise the full narrative corpus and their deeper interpretations, have been reported in detail in two of our articles (Zambetti et al., 2026; Zambetti et al., in press). In the present study, we chose to report only the findings from the 10 narratives of formal caregivers and from the 41 narratives of informal caregivers, to maintain consistency with the study aims described above.

## 4. Results

### 4.1. Villa dei Cedri’s formal and informal caregivers

**Figure 1.**
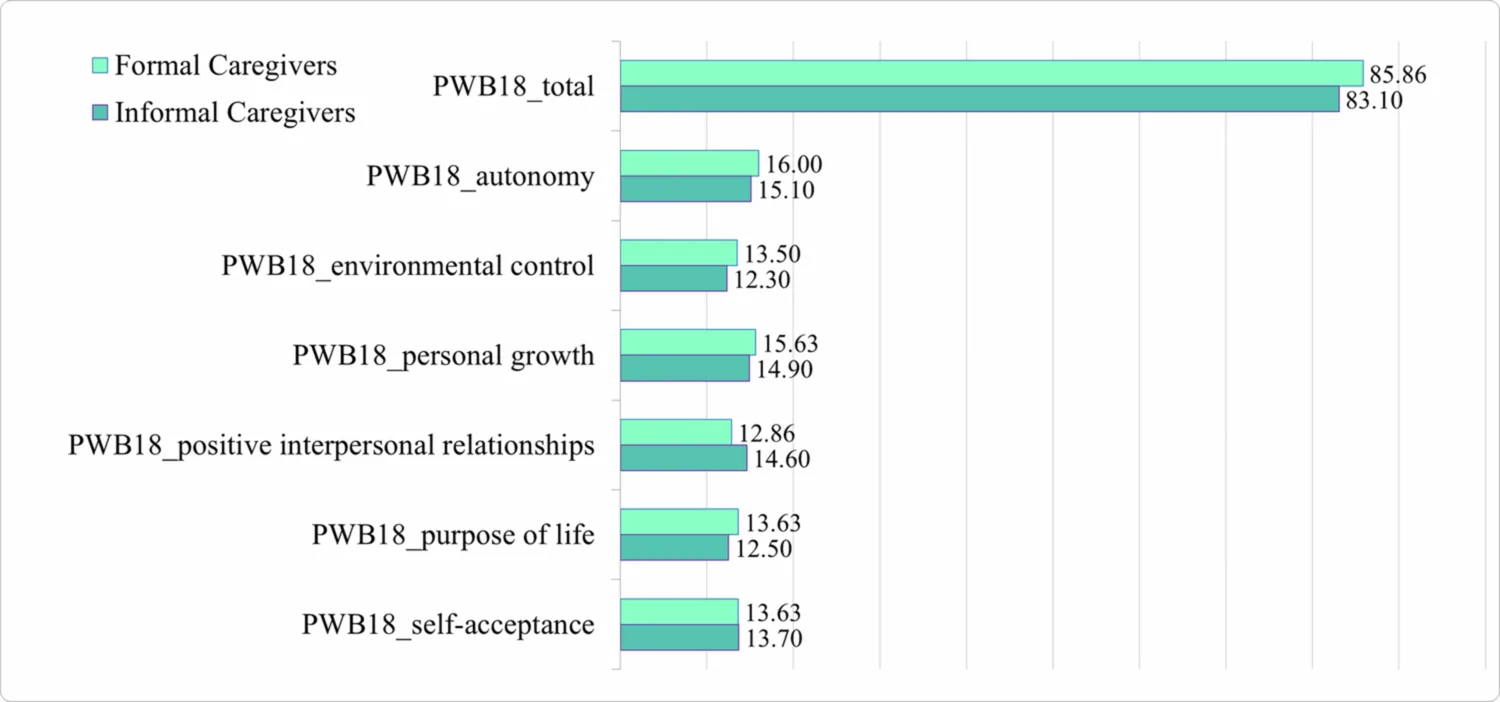
Formal and informal caregivers’ mean scores of the psychological well-being scale and its dimensions.

The non-parametric tests comparing the well-being of formal and informal caregivers found no differences between paid and family care. However, this may be due to the small sample size. Both groups show high psychological well-being across the total score and six subscales, as shown in Figure 1, with higher scores indicating greater well-being. The total scale has a maximum score of 108, and each dimension can score up to 18 (Ruini et al., 2003).

Both formal caregivers (*M =* 85.86; *SD =* 9.026) and informal caregivers (*M =* 83.10; *SD =* 11.435) exhibit high overall psychological well-being, as reflected in their elevated scores across the six scale dimensions. High scores on the autonomy scale suggest that both groups, formal caregivers (*M =* 16.00; *SD =* 1.852) and informal caregivers (*M =* 15.10; *SD =* 2.514), are confident, independent individuals who resist social pressures and evaluate themselves based on their own standards (Ruini et al., 2003). Similarly, high scores in environmental control (formal caregivers: *M =* 13.50; *SD =* 2.828; informal caregivers: *M =* 12.30; *SD =* 2.214) indicate that they feel capable of managing their surroundings and leveraging opportunities to meet their needs (Ruini et al., 2003). Both groups also show high scores in personal growth (formal caregivers: *M =* 15.63; *SD =* 1.996; informal caregivers: *M =* 14.90; *SD =* 2.961), reflecting ongoing development, openness to new experiences, and a drive toward self-improvement (Ruini et al., 2003). Positive interpersonal relationships are well-developed, with formal caregivers scoring (*M =* 12.85; *SD =* 3.805), and informal caregivers (*M =* 14.60; *SD =* 3.062), indicating trust, empathy, and intimacy (Ruini et al., 2003). The participants also report a high purpose of life (formal caregivers: *M =* 13.63; *SD =* 2.264; informal caregivers: *M =* 12.50; *SD =* 4.353), signifying clarity in life goals and the ability to find meaningful significance in past and present experiences (Ruini et al., 2003). Finally, self-acceptance is also rated highly, with a mean score of 13.63 (*SD =* 1.996) for formal caregivers and a mean score of 13.70 (*SD =* 2.946) for informal caregivers, demonstrating positive self-regard and awareness of strengths and weaknesses (Ruini et al., 2003). Overall, these findings are consistent across the measures.

**Figure 2.**
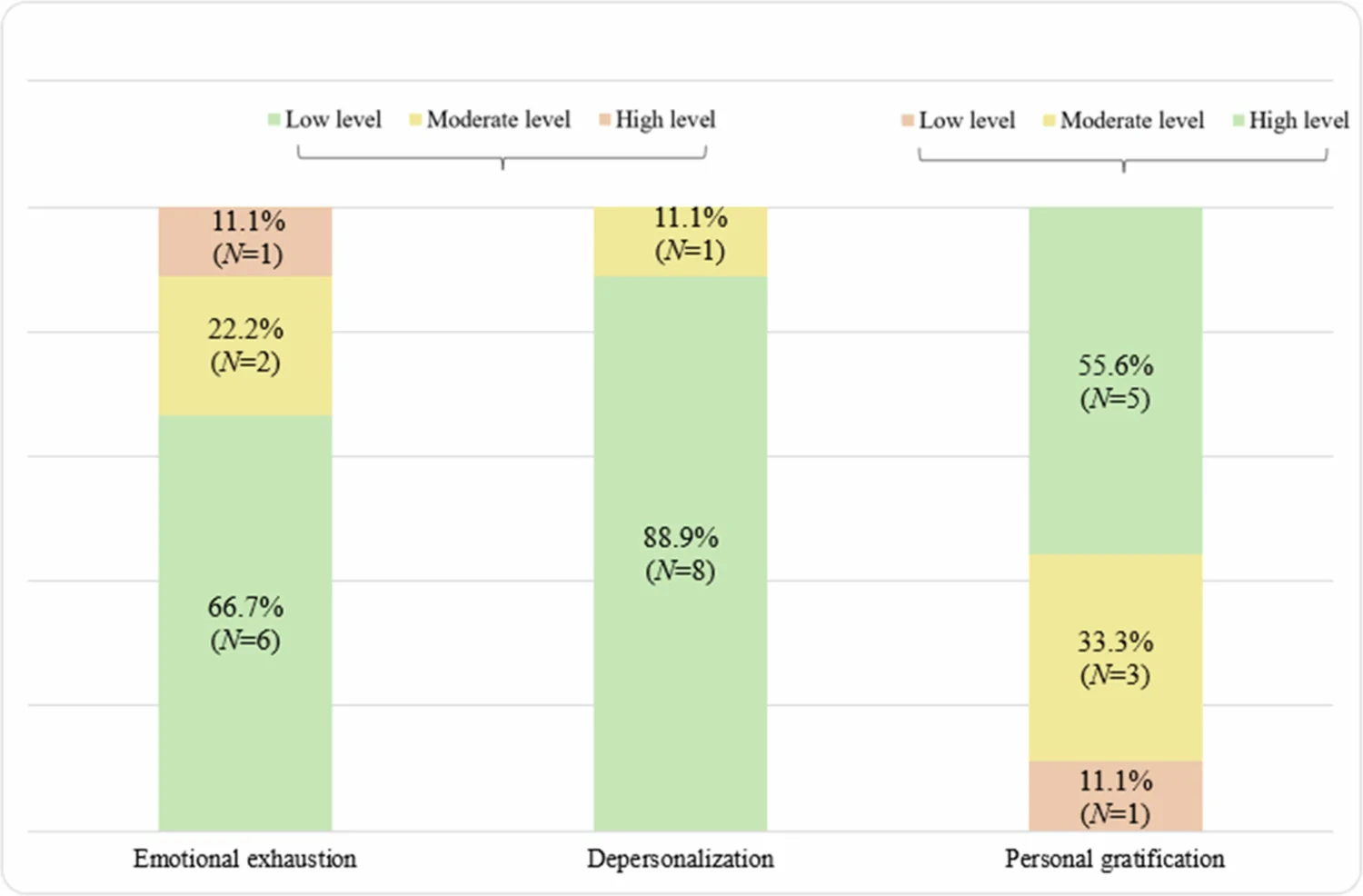
Formal caregivers’ risk of burnout in the three dimensions of the Maslach Burnout Inventory.

Consistent with these results, both formal and informal caregivers exhibit great mean scores in burnout and burden dimensions. Concerning formal caregivers, and so the burnout which could result from paid care, they show low emotional exhaustion (*M* = 16.67; *SD* = 10.087) and depersonalization (*M* = 2.56; *SD* = 2.186), and moderate levels of personal gratification (*M* = 29.11; *SD* = 11.667). These results indicate that healthcare operators in Villa dei Cedri’s ALS unit experience low levels of emotional detachment and disinterest concerning work and other people, together with a moderate perception of their competence and desire for success in collaboration with others. All these results suggest a lower risk of burnout (Maslach et al., 1997). To better assess this burnout risk, the levels of risk within each dimension were classified as low, medium, or high (Maslach et al., 1997). The results are shown in Figure 2.

**Figure 3.**
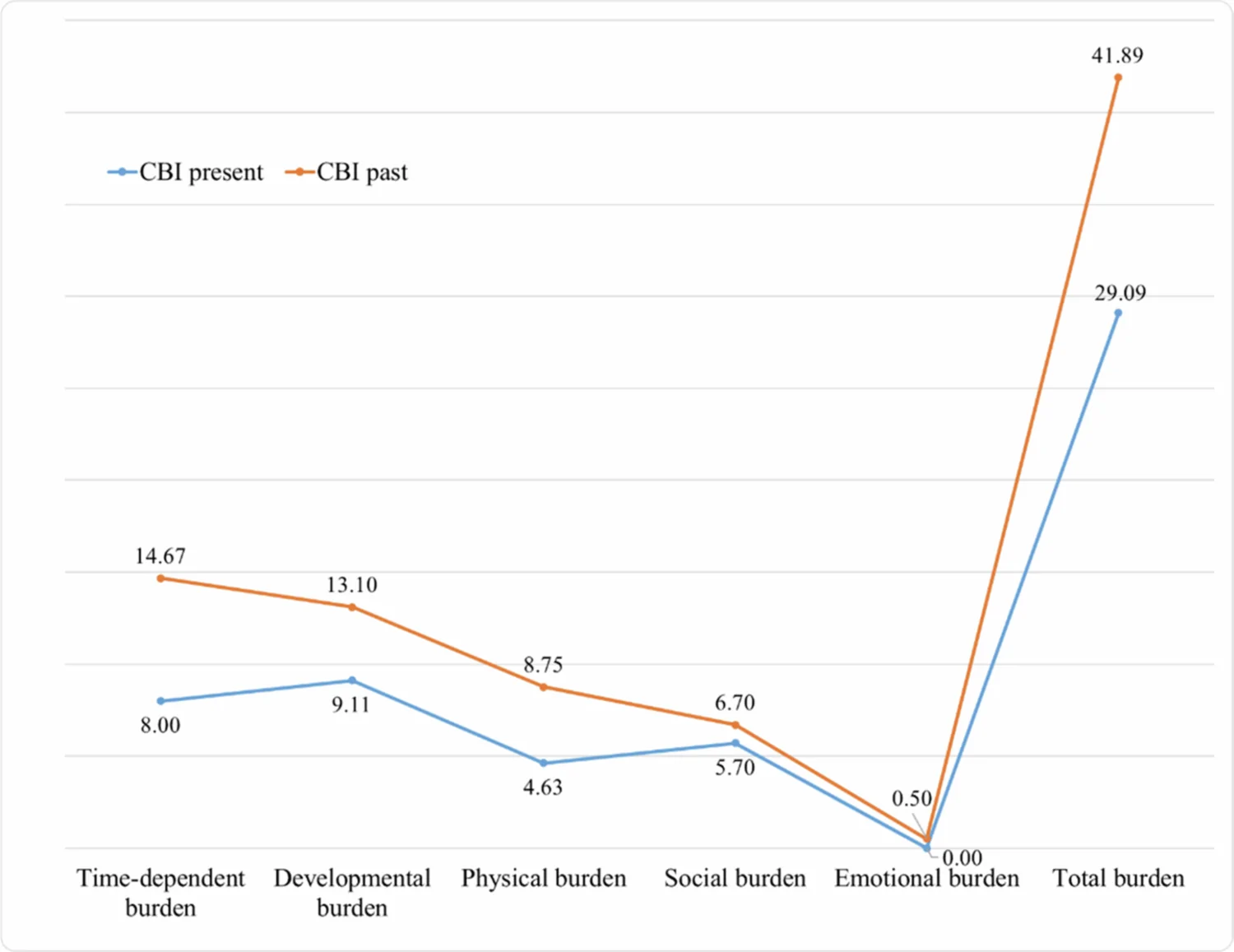
Informal caregivers’ mean scores of the Caregiver Burden Inventory and its dimensions, in past (orange line) and present (blue line) situations.

**Figure 4.**
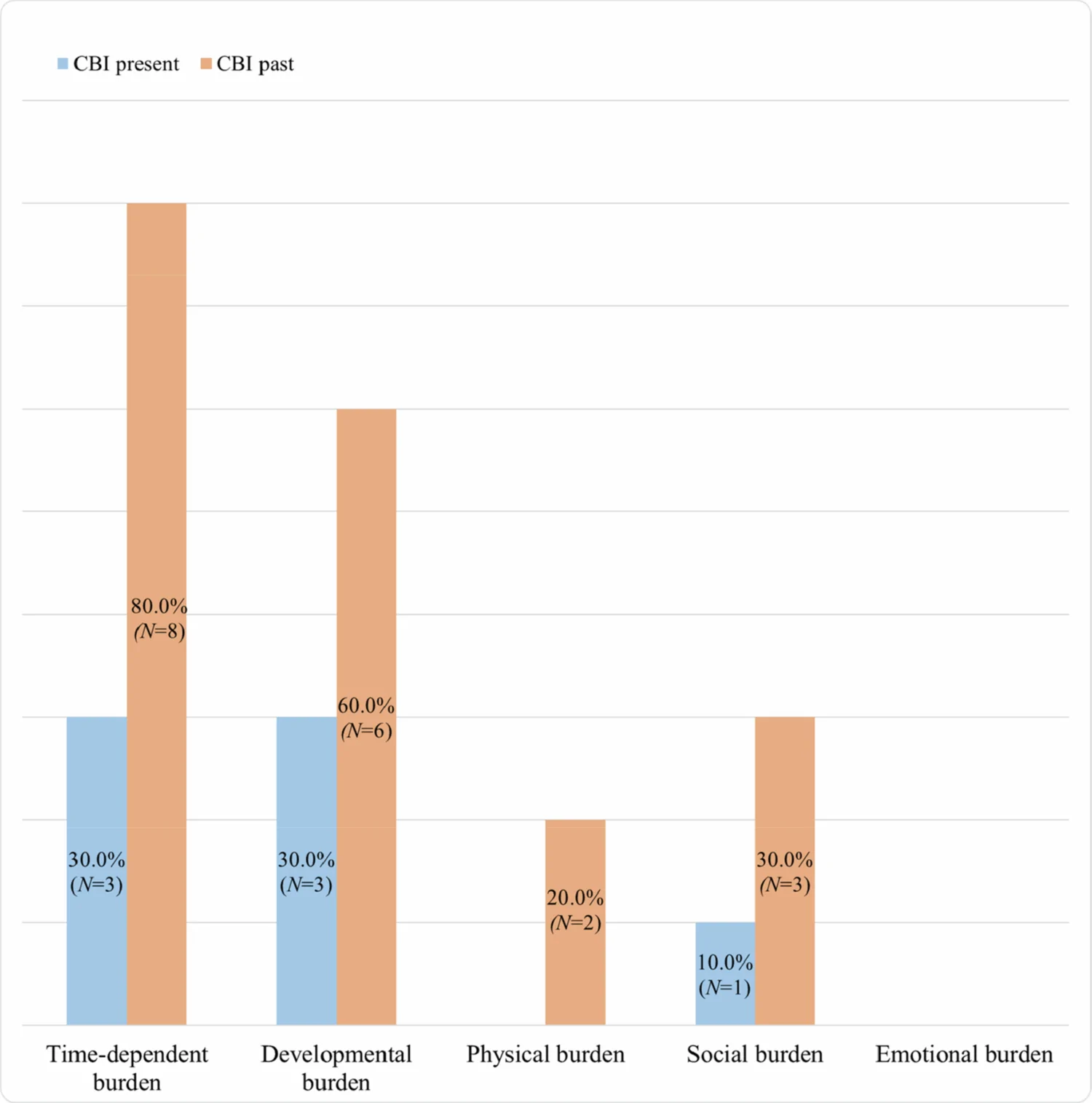
Informal caregivers’ risk of burden in the five dimensions of the Caregiver Burden Inventory, in past (orange line) and present (blue line) situations.

Most formal caregivers have a low risk for burnout related to emotional exhaustion (66.7%; *N =* 6) and depersonalization (88.9%; *N =* 8). They also exhibit a low risk of feeling burnout caused by low personal gratification (55.6%; *N =* 5). We think that these specific results could be influenced by the work and environmental context of Villa del Cedri. Concerning informal caregivers, and so the burden which could result from the family care, we investigated this dimension both in the past (when the patient was still living at home) and in the present (with the patient residing at Villa dei Cedri), and, due to the limited sample size, Wilcoxon Signed Rank Tests with correlated samples were conducted for each pair of variables on the present and past dimensions to determine the presence of statistically significant differences between the two time frames. The results are shown in Figure 3 (mean score) and Figure 4 (burden risk, based on the mean cut-off of 2.5 (Novak and Guest, 1989)).

In general, CBI past scores are higher than present ones, and a significant difference emerges in the dimension of physical burden (*W* = 26.500, *p* = .034). Regarding Figure 4, in general, it appears that for each of the dimensions, except emotional burden, family members who were at risk of excessive burden were at a higher percentage in the past than in the present. In the past, 80.0% (*N* = 8) of informal caregivers were in the risk category for time-dependent burden; in the present, there are only 3 (30.0%) family members. A similar situation occurs in the developmental burden, where in the past 6 (60.0%), informal caregivers were at risk, while in the present, the percentage has dropped to 30.0% (*N* = 3). Two (20.0%) informal caregivers in the past were at risk of physical burden. Regarding social burden, 3 family members (30.0%) were at risk in the past, while, in the present, only one person (10.0%) is in this situation. No one was in the risk category of emotional burden either in the past or in the present.

These preliminary results begin to give us some interesting information about the possible implications for psychological, physical, and emotional well-being between being a caregiver paid to do this work, and being a family caregiver who finds himself or herself in this situation without choice.

### 4.2. Online testimonies

From the previous analysis conducted, five primary themes concerning ALS have emerged: disease onset, difficulties, clinical examinations, coping strategies, and needs. These themes can be mapped onto four broader dimensions of ALS-related communication: disease management, assistance, support, and effort. While the themes represent explicit narrative elements, the dimensions capture the implicit axes of communication and can be generalized across various discursive contexts related to ALS. Both themes and dimensions are embedded within a broader socio-cultural framework that shapes the processes of reality construction. Each dimension is described along a continuum defined by two opposing poles, whose disequilibrium could generate specific effects (Cordella et al., 2014; Greco, 2016).

Overall, narratives produced by formal caregivers appear less likely than those of family members to explicitly report their own needs or the challenges encountered in the context of their professional paid care activities. On the other hand, family members’ narratives tend to emphasize both their own needs and those of patients, particularly concerning the ALS experience. A more detailed and theme-specific analysis of these aspects, organized according to the identified themes and dimensions, is presented below, with particular attention to the dominant aspects within each discourse.

#### 4.2.1. Themes

##### 4.2.1.1. Disease onset

The theme centered on the disease’s onset primarily concerns the feelings and emotions expressed by family caregivers when ALS is first discovered and diagnosed. Although it is mainly a theme relevant to patients’ families, important aspects also appear in the stories of formal caregivers.

In their stories, informal caregivers (cf. family members) recount how ALS first showed itself through initial symptoms of their loved one and how they reached the diagnosis. The stories are very detailed, often including specific spatial and temporal references and symptom details related to the start of ALS, as in these stories.

*Tutto è incominciato alla fine di luglio del 2010. Toni stava attraversando un periodo di depressione a causa di un lutto in famiglia e incominciava ad avere fascicolazioni ai muscoli pettorali, labilità al pianto e stanchezza. Il medico all’inizio gli ha prescritto antidepressivi che lui non sopporta, ne prova 4 tutti con gli stessi risultati, addirittura gli provocavano idee suicide. Poi è stato necessario il ricovero, a novembre, e lì è arrivata la diagnosi: Malattia del Motoneurone. Insomma Toni ha la SLA. Di conseguenza è cominciata anche la terapia con Rilutek. È lì, davanti a quella diagnosi, che la vita è cambiata.*

(It all started at the end of July 2010. Toni was going through a period of depression due to a family bereavement and began to have fasciculations in his pectoral muscles, lability to crying, and fatigue. The doctor at first prescribed antidepressants that he could not stand; he tried 4 of them all with the same results, even causing him suicidal thoughts. Then hospitalization was necessary, in November, and there came the diagnosis: motor neuron disease. In short, Toni has ALS. As a result, treatment with Rilutek also began. It was there, in front of that diagnosis, that life changed.)

While the narratives of family caregivers often refer to themselves, the patient (as well as family members) and their own experiences, including emotional ones, formal caregivers mostly report technical aspects related to the physical and psychological manifestations typical of the onset of the disease. This theme, present in both groups of formal and informal caregivers, is closely related to the following theme of difficulties.

##### 4.2.1.2. Difficulties

The difficulties that emerge in the narratives of formal and informal caregivers involve physical, psychological, and emotional aspects.

Once again, family members tend to include very personal aspects in their stories, which relate to the difficulties perceived by them as caregivers of ALS patients:

*Il mio punto di vista è quello di una figlia, non di una persona affetta da SLA e il mio dolore è senz’altro diverso da quello di mio padre, è un dolore ‘esterno’, ma forte e devastante, perché quando un familiare si ammala, si ammala tutta la famiglia.*

(My point of view is that of a daughter, not a person with ALS, and my pain is certainly different from my father's; it is an ‘external’ pain, but a strong and devastating one, because when one family member gets sick, the whole family gets sick.)

But also the difficulties they perceive to be present in their loved one who has the disease:

*Ti ha consumato lentamente, tagliandoti piano piano le capacità di camminare, di controllare i movimenti del tuo corpo, alla fine di parlare, di mangiare e di respirare.*

*Ti ho perso poco alla volta, lacerandomi il cuore nel vederti sempre più in difficoltà.*

(It slowly consumed you, slowly cutting off your ability to walk, to control your body movements, eventually to speak, to eat, and to breathe.

I lost you little by little, tearing at my heart as I saw you struggling more and more).

Patients and family caregivers, in the daily difficulties that change in form and intensity as ALS progresses, thus find themselves living a life constantly accompanied by a variety of questions, including and especially of a practical-organizational nature, but which have an emotional impact:

*Pensavo tutto insieme in un secondo:*

*Come lo trasporto?*

*Come lo proteggo?*

*Come lo faccio transitare nelle sale d’attesa piene di gente?*

*Come faccio a non farlo stancare?*

*Come faccio a trasportare lui, la NIV (ventilatore non invasivo), il borsone della documentazione clinica da sola e senza la sedia a rotelle (che non abbiamo perché papà, fortunatamente, cammina)?*

*Occhiali appannati, sudore sulla fronte, disperazione.*

*Mille domande e nessuna risposta.*

(I was thinking altogether in a second:

How do I transport it?

How do I protect it?

How do I get him through the waiting rooms full of people?

How do I keep him from getting tired?

How do I carry him, the NIV (non-invasive ventilator), the duffel bag of clinical documentation alone and without the wheelchair (which we don't have because dad, fortunately, walks)?

Fogged glasses, sweat on forehead, despair.

A thousand questions and no answers.)

Formal caregivers’ stories, again, direct their focus primarily to the difficulties of the family and the patient, almost neglecting those difficulties they themselves might experience as formal caregivers.

The personal, physical, and emotional dimensions of difficulty experienced by formal caregivers thus seem to be put on the back burner, perhaps because it is perceived as distant and inferior (in intensity and severity) to that experienced by patients and family members, and perhaps more important to emphasize.

##### 4.2.1.3. Medical examinations

The theme concerning the medical aspects and examinations necessary for ALS diagnosis and subsequent follow-ups is most present in informal caregivers, in the form of technical explanations of the various procedures and the related emotional consequences. The recurring element in all the narratives is that of the perception of these visits as dilated in an infinite amount of time, a real ‘*calvario*’.

This aspect could be explained by the fact that family members are the ones who take on this aspect as well, accompanying the ALS patient to the different visits, thus perceived as an additional burden (physical and emotional), as also revealed in the quantitative results related to the CBI.

*Colpiscono ogni singolo aspetto della vita di chi ne è affetto e di chi gli sta attorno, diventando il centro del loro mondo, e condizionando ogni momento della loro giornata, ed ogni loro scelta, anche a lungo termine.*

(They affect every single aspect of the lives of those affected and those around them, becoming the center of their world, and affecting every moment of their day, and their every choice, even in the long term.)

This more technical aspect, directly related to medical examinations, does not emerge in the narratives of social workers. This, perhaps, is because, as mentioned earlier, formal caregivers’ narratives are more oriented toward highlighting the suffering and difficulties, but also the needs, of patients and families. The technical and medical procedure part, therefore, could be seen as a superfluous element in narrating one’s professional experience with ALS.

##### 4.2.1.4. To cope

In light of the aspects of physical and emotional difficulties and the necessary reorganization of family and personal life, another important theme that emerges concerns coping with ALS and its challenges.

Within the narratives of family members, the theme of ‘to cope’ is predominantly narrated by referring to everything new they learn to do, to manage even the smallest aspects of daily life:

*Occorre attivarsi per adeguare il cuore e, operativamente, la casa.*

*Il confort diventa la parola chiave. Tutto deve essere finalizzato ad affrontare giornate di quiete a giornate di potenziale allarme…*

(It is necessary to take action to adjust the heart and, operationally, the home.

Comfort becomes the keyword. Everything must be aimed at dealing with days of quiet to days of potential alarm…)

The common elements in these narratives turn out to be love, the strong feeling of union to one’s family, social closeness, and faith or spirituality that gives one the strength to go forward.

In contrast, the coping-related element that emerges in the formal caregivers’ narratives is more related to the desire to find strategies for early diagnosis and treatment. The narratives thus refer to constant activation in research as a strategy to overcome (or hope to do so in the more or less near future) ALS.

##### 4.2.1.5. Needs

*Sono arrivati il panico, la fame di sapere a fonte della scarsa informazione, e la paura.*

(There came panic, hunger for knowledge at the source of poor information, and fear.)

With this brief excerpt, some of the main needs of family members of an ALS patient emerge. The informational need, thus related to knowledge of the disease, is not so much from a technical point of view, when focused more on what it practically entails in the here and now and in the future. The need to manage negative emotions, which inevitably arise from the initial lack of information. And, in general, the underlying need to be supported, as one is afraid to be left alone to manage and experience the disease of one’s family member. These needs are very much related to the different implicit dimensions of communication, which will be presented in the following paragraphs.

Family members, therefore, report multidimensional needs, needed by both them and their loved ones with ALS:

*Perché la cura alla persona con malattia cronica degenerativa di terzo livello è, su tutto e prima di tutto, relazione di condivisione, ascolto, empatia e rispetto delle routine basilari, residuali, vitali.*

*Senza cibo si dura un po’, senza acqua di meno, senza aria per niente. Salvo barattare la propria voce, i propri gusti culinari, il ritmo del proprio respiro e farsi circuiti, sacche, sonde, prolungamenti di pompe.*

(Because caring for the person with chronic degenerative third-level disease is, above all and above all else, a relationship of sharing, listening, empathy, and respect for basic, residual, vital routines.

Without food one lasts a little, without water less, without air for nothing. Except trading one's voice, one's culinary tastes, the rhythm of one's breathing, and making oneself circuits, bags, probes, and pump extensions.)

Formal caregivers, on the other hand, once again dwell on highlighting what the needs of families and patients are, at the expense of highlighting their personal needs.

As with previous themes, social and healthcare workers are key in raising awareness of ALS and its impact on mental health. Those with the disease emphasize the value of connecting with other professionals and patients to optimize caregiving, but also highlight the need for psychological support to cope with grief.

#### 4.2.2. Dimensions

##### 4.2.2.1. Disease management

Disease management is the first implicit dimension of communication that emerges in the narratives of paid and family caregivers. It is arranged along a continuum that involves emotional management of ALS on one side and physical management on the other. These two sides must be balanced with each other to ensure comprehensive caregiving, whether provided by family or professionals.

In the narratives of family members, the emotional management of ALS tends to emerge more, the constant struggle with this disease:

*La SLA è una malattia crudele, e ancora oggi quasi del tutto sconosciuta. E la lotta delle famiglie dei malati è molto spesso uno sforzo solitario. Malattie come questa colpiscono molto più della semplice salute.*

(ALS is a cruel disease, and still almost completely unknown. And the struggle of the families of sufferers is very often a lonely effort. Diseases like this affect far more than just health.)

However, there is no shortage of more practical elements, typical of physical disease management, enacted in the everyday lives of patients and family members:

*A Padova ci hanno dato degli integratori perchè ha perso 12 kg, io ho aggiunto estratti liofilizzati di frutta e verdura anche se mangia normale come sempre, e da un paio di settimane anche minerali antiossidanti e gocce omeopatiche, prescritte dallo psicologo per sollevarlo dalla labilità al pianto.*

(In Padova, they gave us supplements because he lost 12 kg, I added freeze-dried extracts of fruits and vegetables even though he eats normally as usual, and since a couple of weeks, also antioxidant minerals and homeopathic drops, prescribed by the psychologist to relieve him from lability to crying.)

In formal caregivers, the narrative focus is predominantly on the difficulties of coping, both physically and emotionally, with ALS, encountered by patients and family members:

*In quei brevi momenti racconta le inevitabili difficoltà del loro rapporto, difficoltà che a volte sembrano macigni, ma sono solo incomprensioni, parole di troppo, stanchezza… La quotidianità dei due coniugi è messa a dura prova dalla malattia e la pazienza di Diego e della stessa Luciana sembra infrangersi contro un muro di gomma che rimbalza da una parte all’altra.*

(In those brief moments, he recounts the inevitable difficulties of their relationship, difficulties that at times seem like boulders, but are only misunderstandings, words too many, fatigue... The daily routine of the couple is put to the test by the disease, and the patience of Diego and Luciana herself seems to break against a rubber wall that bounces from one side to the other.)

Little emerges regarding their personal experience of managing the disease, which is mostly physical and pharmacological.

##### 4.2.2.2. Assistance

Assistance is the second implicit axis of communication that emerges from these testimonies, and it is divided between technological and relational. Again, the two elements should be well-balanced in order to provide optimal and efficient caregiving for the patient.

Regarding technological aspects, their importance for a better quality of life of the ALS patient emerges in both informal and formal caregivers. Indeed, the patient becomes more and more dependent on people but also on technology (e.g. Assisted Mechanical Ventilation, cough aspirator, food canula, etc.), which allows him/her to survive.

*SLA […] orizzonte di vita all’insegna della progressiva dipendenza dagli altri, dai farmaci, dagli ausili, dalle macchine.*

*SLA, Senza La Autonomia.*

*Perché per gradi o repentinamente si perdono funzioni e abilità che inchiodano alla sedia, chiamata comoda—comoda per chi?—che costringono alla poltrona, che bloccano a letto.*

*SLA, Senza La Autonomia.*

*Sì, proprio così, perché intralcia la parola sino a inibirla, ostacola la deglutizione sino a bloccarla, riduce il movimento sino ad azzerarlo, soffoca il respiro sino a impedirlo.*

*Limiti pesanti che innescano un processo di non ritorno.*

*Non c’è prevenzione, non c’è vaccino, non c’è guarigione, c’è solo da scendere a patti, senza eroi e senza santi. La dignità a baluardo.*

(ALS [...] horizon of life under the banner of progressive dependence on others, on drugs, on aids, on machines.

ALS, Without Autonomy.

Because by degrees or abruptly one loses functions and abilities that nail one to the chair, called comfortable - comfortable for whom? – That force on the chair, that lock in bed.

ALS, without the autonomy.

Yes, that's right, because it hinders speech to the point of inhibiting it, hinders swallowing to the point of blocking it, reduces movement to the point of zeroing it, and chokes breath to the point of preventing it.

Heavy limitations that trigger a process of no return.

There is no prevention, no vaccine, no cure; there is only to come to terms, without heroes and without saints. Dignity as a bulwark.)

Central to both caregiving roles, however, remains the human and relational aspect inherent in different forms of assistance. Only through this, in fact, is it possible to restore some dignity and humanity to the patient, and to continue to make him or her feel part of a community. The relational aspect, as well explained by both family members and healthcare workers, emerges in the support of friends, relatives, and ALS associations, but also in the therapeutic professional support, where a new relationship is established that can be of assistance in overcoming the different critical stages of the disease, and integrating this traumatic event into one’s life story.

*[…] un intervento psicoterapeutico per queste persone, laddove le condizioni cliniche lo permettono, [...] consentendo alla persona la ricostruzione della trama della sua storia, la tessitura dell’esperienza frammentata e il recupero della sua identità integrandola con quella attuale.*

([…] a psychotherapeutic intervention for these people, where clinical conditions permit, […] enabling the person to reconstruct the fabric of his history, weave the fragmented experience, and recover his identity by integrating it with his current one.)

##### 4.2.2.3. Support

The third implicit axis of communication is support, social, and self-directed. Social support is perceived as an essential element, especially in ensuring a sense of belonging to the community, both of patients and family caregivers. This aspect emerges in both the narratives of family caregivers and paid caregivers. The first ones focus on their own needs for social support, and those of their loved ones with ALS

*Immediatamente compresi che il sostegno degli altri, il supporto materiale e pratico sarebbe stato fondamentale per affrontare questa patologia, che bisognava rendere ancora più forti i legami con le persone che ami per proteggersi tutti dalla solitudine e dallo sconforto, che spesso ti prende.*

(Immediately, I understood that the support of others, the material and practical support, would be crucial in dealing with this disease, that you need to make the bonds with your loved ones even stronger to protect all of you from the loneliness and despondency, which often takes you.)

The latter, on the other hand, once again neglect their own direct experience and support needs, in favor of what they perceive as necessary support that needs to be done toward patients and their families, telling the life stories of patients.

On the other hand, self-directed support is an element that only emerges in the testimonies of family members, who indicate the importance of gaining strength daily to move forward in the fight against their loved one’s disease. This pole is very emotionally charged, and it is especially enacted when the familiar caregiver must move on after the death of the person with ALS.

*Ho ancora sul mio petto il tuo ultimo respiro e lo porterò gelosamente per sempre sul mio corpo, nelle mia mente, nel mio cuore. […] Mi consolerà il ricordo del tuo volto solare e mi sosterrà tutto l’amore, la gioia e la forza che mi hai dato.*

(I still have your last breath on my chest, and I will jealously carry it forever on my body, in my mind, in my heart. [...] I will be comforted by the memory of your sunny face and sustained by all the love, joy, and strength you gave me.)

##### 4.2.2.4. Effort

Finally, the last axis of implicit communication concerns effort: personal and institutional. In both family caregivers’ and paid caregivers’ narratives, effort is seen as an essential element in making it possible to combat, in the present and in the future, ALS. The components of such narratives are both emotional (for the most part) and have practical implications and examples of actions to be implemented.

Among social and health workers, the importance of continuing to do research (institutional effort) emerges on the one hand, and on the other hand, the need to actively fight even in the present for families who struggle and have struggled (personal effort):

*Il silenzio a volte urla e quel giorno mi ha trasmesso la voglia di continuare ad andare avanti e a lottare. Le lacrime non riesco a trattenerle. Sono per Nicola, ma anche per Pietro, per Angelica, per Anna, per Vincenzo…per tutti quelli che ci hanno lasciato dentro la convinzione che tutto si può fare meno che lasciarsi sopraffare dalla SLA.*

*Così indosso ancora una volta la mia divisa da soldato Ryan, sperando di poter tornare un giorno a casa e di portare con me tutti i malati di SLA finalmente guariti e le loro famiglie.*

*E rendo onore ad un altro grande guerriero che porteremo sempre nel cuore.*

(Silence sometimes screams, and that day conveyed to me the will to keep going and fighting. The tears I cannot hold back. They are for Nicola, but also for Pietro, for Angelica, for Anna, for Vincenzo...for all those who have left us with the conviction that everything can be done, rather than letting ALS overwhelm us.

So I wear my uniform once again as Private Ryan, hoping to one day return home and take with me all the ALS patients who have finally been cured and their families.

And I honor another great warrior who we will always carry in our hearts.)

Family members, in their narratives, combine personal and institutional effort: only in this union of forces do they see the possibility of raising awareness and progress with research, thanks to both the efforts of professionals and donations from citizens. On the one hand, therefore, personal effort lies in telling one's story and spreading it; on the other hand, however, it is important that an institutional effort of ALS recognition and research also takes place:

*Se raccontandovi la mia esperienza e quella di mia madre c’è un messaggio che posso lanciare per farvi riflettere, per farvi considerare il problema, e per farvi lottare per migliorare le condizioni dei malati e delle loro famiglie, allora lo farò.*

*Se volete davvero mantenere viva la sua memoria, quello che veramente vi chiedo è, in cuore vostro, di non dimenticare mai che il problema della SLA e delle malattie rare esiste. Di non sottovalutare mai l’impatto che queste hanno su un numero di vite più grande di quello che possiate immaginare. E di non pensare mai di non avere il potere di cambiare le cose.*

*La solidarietà, l’impegno, il finanziamento della ricerca, forse, un giorno, potranno fare in modo che nessuno soffra come ha sofferto mia madre.*

(If by telling you about my experience and my mother's experience there is a message I can send out to make you think, to make you consider the problem, and to make you fight to improve the conditions of sufferers and their families, then I will do so.

If you really want to keep her memory alive, what I really ask of you is, in your heart, to never forget that the problem of ALS and rare diseases exists. To never underestimate the impact they have on more lives than you can imagine. And to never think that you do not have the power to change things.

Solidarity, commitment, and funding for research, perhaps, one day, they can make sure that no one suffers as my mother suffered.)

## 5. Discussion

The study examined what ‘paid care’ or ‘family care’ means for people with ALS, focusing on the impact on well-being, burnout, and burden. We analyzed data from Villa dei Cedri and people who published online their caregiving journeys.

The study revealed that formal and informal caregivers have high psychological well-being scores and low burnout. These scores indicate high confidence, independence, resistance to social pressures, and self-regulation. High scores on the environmental control scale show that caregivers can manage their environment and take advantage of opportunities. High scores on the personal growth scale indicate ongoing development and openness to new experiences. Both groups reported positive relationships, trust, empathy and intimacy. Family caregivers and paid caregivers reported clear life purposes, and high scores on the self-acceptance scale indicated positive self-perception. Specifically for family (or informal) caregivers, it then emerges that the highest burden scores are found when the patient was still living within the home walls. This burden, in fact, seems to be significantly lower when the patient is placed in a residential health care facility, with 24-hour professional care. This result suggests to us how daily care and support carried out by medical personnel are of necessary importance and can help decrease, in family caregivers, the perception of physical, time-dependent, social, and developmental burden.

Specifically for formal (or paid) caregivers, low levels of emotional exhaustion and depersonalization, and moderate levels of personal gratification then emerge. Caregivers in the Villa dei Cedri ALS unit thus experience low levels of emotional detachment and disinterest in their work and interactions with others, along with moderate perceptions of their own competence and a desire for success in working with others. All these findings suggest a lower risk of burnout. These findings, which differ from previous studies (Baiocco et al., 2004; Karabuga Yakar et al., 2023) in the literature conducted on formal caregivers, could result from the private work context in which they are placed, which is surrounded by a large portion of green environment, set in a park, and structural and architectural elements that could be a protective factor of burnout. This factor represents one of the limitations of the research, along with the small sample size. Neither of these factors allows for the generalizability of the data, but rather enables a specific preliminary exploration within the specific residential context of Villa dei Cedri. However, further research with a larger sample and in different occupational health settings is needed to confirm or disconfirm these findings.

Discourse analysis shows many testimonies incorporate an emotional element, conveying to the reader what ALS entails. This emerges when formal caregivers talk about ALS patients, often including personal recollections or emotional considerations about the situation with the patient and their family. These narratives emphasize technical aspects, such as diagnosis and examinations, and supportive technologies, while also highlighting the needs and wishes expressed in interactions with patients and families. Research is needed to understand the emotions of these caregivers.

The aspects described above also appear in family testimonies, mostly about personal struggles, needs, and requests for help. These testimonies explore the multiple aspects and meanings of ALS, a topic that is still little studied. The analysis examined the narratives in detail to foster understanding of the thoughts and emotions of people facing this disease. The two research studies presented, although developed in different contexts and through different methodologies, similarly highlight the importance of context in supporting professional and family caregivers. In particular, the study conducted with Villa dei Cedri suggests that a natural environment can positively impact burnout, stress, and well-being of caregivers. It also suggests how institutionalization is a supportive element in reducing the caregiving burden of family members. Therefore, it is essential to investigate these aspects to shed light on the protective factors in caregiving.

In parallel, the analysis of online testimonies highlights a strong dedication and continuity in commitment against the disease. These narratives reveal the existence of coping strategies, altruistic motivations, and a collective vision of caring for each other.

In this sense, the integration between a supportive environmental context and a network of shared meanings built through storytelling emerges as a key element in promoting the well-being and resilience of paid and family caregivers, helping to reduce the risk of burnout and burden (Charon, 2008, 2017). Considering this evidence, it seems appropriate to initiate further research, such as through interviews and focus groups targeting these figures, to gain a deeper and more nuanced understanding of their experiences within their specific socio-cultural context. Only through this perspective will it be possible to design training and support pathways adapted to different work and non-work environments, capable of enhancing both personal and organizational characteristics, to enhance personal resources and promote caregivers’ overall well-being.

## Ethical considerations, consent to participate, and consent to publish

For the first part of the study, conducted in Villa dei Cedri, the research was approved by the Lombardy 2 Regional Ethics Committee at its meeting on December 11, 2024 (protocol code: L2-270). The research was also conducted following the ethical principles outlined in the Declaration of Helsinki. Each participant included in the study provided their informed consent to participate in the research and to publish.

For the second part of the study, conducted on online testimonies, this is a study that does not directly involve people but analyzes testimonies spontaneously produced and voluntarily posted online, accessible to anyone with an Internet connection. Since no participants were recruited, and no sensitive information is present, the study does not require ethical approval. Following a preliminary review conducted via the platform of the Committee for Integrity and Research Ethics of the University of Bergamo, the research project was deemed ‘not applicable’ for evaluation by the Committee, as (to quote the response received) it does not present any ethical or legal issues that require a formal opinion. This outcome is consistent with the terms of the Regulations of the Committee for Integrity and Research Ethics of the University of Bergamo (Article 3, paragraph 1, letter a, and paragraph 2), which establishes that only researches involving individuals and/or with significant ethical implications are subject to ethical evaluation, excluding activities that do not implicate the direct involvement of human subjects, the use of sensitive personal data or experimental interventions, and research that takes the form of literature reviews or theoretical and methodological analyses. This regulation also complies with the Regulation of the Territorial Ethics Committees (CET) of the Lombardy Region (Articles 3 and 4), which identifies the competences of the CET in the evaluation of clinical trials, observational studies, pharmacological studies, and research involving different types of interventions and research that actively involve people. Therefore, in light of the official outcome of the preliminary review and the current regulatory framework, we can confirm that the project does not need to be evaluated by the Ethics Committee. However, the research was conducted following the ethical principles outlined in the Declaration of Helsinki, also making written testimonies posted online anonymous.

## Data Availability

Research data is available upon request to the authors. We also declare that: (a) Institutional Review Board Statement: The study was conducted in accordance with the Declaration of Helsinki and was approved by an Institutional Review Board/Ethics committee. See details under Methods. (b) The study received an exemption from an Institutional Review Board/Ethics committee. See details under Methods.

## Endnotes

^1.^
The formal caregivers (*n* = 9) who voluntarily participated in the study are part of a group of 20 healthcare operators in the ALS unit. Of these: 2 are physicians, 9 are nurses, 7 are OSS, and 1 is a physiotherapist. In total, however, there are 82 healthcare workers at the facility, including 7 physicians, 18 nurses, 52 OSS, and 4 physiotherapists. The facility also has a psychologist who serves all units.

^2.^
As for family members, before the patients were admitted to Villa dei Cedri, they all played an active role in providing care, including caregiving at home. In fact, most of them are adult children who live away from home but played an active role in caring for their parent before he or she entered the nursing home.

^3.^
Themes are explicit representations that emerge in spoken or written discourse; they represent categories of topics mentioned by narrators and are identified during an initial phase of text analysis. The axes of communication, on the other hand, are implicit representations, culturally and socially constructed, that emerge through in-depth discourse analysis.
